# Quadruplicate Synchronous Adenocarcinoma of the Colon with Distant Metastases—Long-Term Molecular Follow-Up by KRAS and TP53 Mutational Profiling

**DOI:** 10.3390/diagnostics10060407

**Published:** 2020-06-16

**Authors:** Emese Sarolta Bádon, Attila Mokánszki, Anikó Mónus, Csilla András, László Damjanovich, Gábor Méhes

**Affiliations:** 1 Department of Pathology, Faculty of Medicine, University of Debrecen, H-4032 Debrecen, Hungary; badon.emese.sarolta@med.unideb.hu (E.S.B.); mokanszki.attila@med.unideb.hu (A.M.); monusne.aniko@med.unideb.hu (A.M.); 2 Department of Oncology, Faculty of Medicine, University of Debrecen, H-4032 Debrecen, Hungary; andras.csilla@med.unideb.hu; 3 Department of Surgery, Faculty of Medicine, University of Debrecen, H-4032 Debrecen, Hungary; dami1960@med.unideb.hu

**Keywords:** colorectal cancer, metastasis, genetic alterations, *KRAS*, next-generation sequencing

## Abstract

Anatomically independent tumor foci represent biologically distinct neoplasias, potentially featured by different progressivity and treatment responsiveness. To demonstrate the biological complexity, a metastatic colon adenocarcinoma patient originally presenting with four independent primary tumors of the right colon half and altogether eight distant metastases was followed by molecular testing. Next-generation sequencing results highlighted the mutational profile of the individual primaries and the dynamics of the different gene variants observed during follow-up. The four primary colon tumors presented with four different *KRAS* genotypes, one of them with a wild-type and three with pathogenic variants, without overlap. These were the following: c.35G > A; p.Gly12Asp with 40.6% variant allele frequency (VAF); c.34G > T; p.Gly12Cys with 16.2% VAF and c.35G > T; p.Gly12Val with 15.1% VAF. In metastatic tumors, with one exception where no mutation was detected, only the *KRAS* c.34G > T; p.Gly12Cys mutation could be detected. *TP53* gene variants were variable in the primary tumors, with a single dominant variant evolving in the follow-up metastases (c.820G > T; p.Val274Phe). Genetic profiling of individually developing synchronous malignancies uncovers the clonal relations of metastatic tumors. NGS gene panels provide a solution to follow the dynamics of key oncogene variants during the course of the disease and greatly contribute to therapy optimization.

## 1. Introduction

Multiple cancer foci are typically associated with hereditary cancer syndromes and are associated with germline gene mutations such as familial adenomatous polyposis or hereditary non-polyposis colon cancer [1,2,3]. However, tumor multiplicity is increasingly common in non-hereditary, sporadic disease, and is associated with soft risk factors, including male gender and advanced age [1,2]. A progressive tumor mass developing in the colon is frequently accompanied by dysplastic adenomas or other forms of precancerous superficial lesions, which may be underdiagnosed by colonoscopy or surgical sampling [4,5]. Moreover, undetected synchronous and metachronous cancer foci may be interpreted as one disease (local recurrence) unless analyzed in detail.

Molecular testing in colorectal carcinomas includes the exclusion of *KRAS*-, *NRAS*- and *BRAF*-activating mutations as a mandatory precondition for the efficacy of anti-EGFR therapy [6,7,8,9,10]. According to the current diagnostic procedure, the analysis of a representative tumor sample is required for the determination of the relevant gene sequences. Pathogenic gene variants are individual and characteristic, and despite variable degrees of heterogeneity, major variants remain stable during dissemination. In general, the mutation profiles of metastases are based on that of the primary cancers from which they originate [11]. The exact clonal origin, further influenced by selection mechanisms, e.g., dissemination or treatment, basically determines survival and resistance to the applied targeted therapy [12,13,14,15].

However, the management of individual distant metastases in case of multiple primaries becomes challenging, especially if the primary foci present with different genetic features. Several studies have evaluated the potential dynamics of multiple genotypes in synchronously presenting colon adenocarcinomas, especially when different *KRAS* genotypes were demonstrated. In our work, we followed the clonality and genetic variation of a quadruple colon cancer consisting of four simultaneous separate colon tumors developing multiple metastases over a four-year period of follow-up. The aim of our study was the retrospective identification of individual colon adenocarcinoma foci by molecular genetic tools and the demonstration of clonal relations between primary tumors and distant metastases. For this purpose, a small next-generation sequencing (NGS) solid tumor gene panel (Illumina MiSeq platform) was used. The NGS results were confirmed using strip-based reverse hybridization assay (Stripassay) and conventional Sanger sequencing.

## 2. Materials and Methods

Altogether, thirteen formaldehyde-fixed paraffin-embedded tissue (FFPE) samples were tested from the same patient diagnosed with colon carcinoma in 2015 and followed until his death in August 2019 at the Department of Oncology, University of Debrecen. All protocols were approved by the authors’ institutional review board for human subjects (IRB reference number: 60355/2016/EKU). One initial colon biopsy sample, four independent primary tumors of the surgically removed subtotal colectomy preparate, and eight metastatic tumor samples from five different time points were collected and evaluated. Hematoxylin and eosin (H&E)-stained slides were reviewed by two pathologists and tumor tissue was selected for analysis with a >20% tumor percentage. Immunohistochemistry for the four most common mismatch repair proteins (MLH1, dilution: 1:50 antibody clone G168-728, Cell Marque, Rocklin, CA, USA; MSH2, dilution: 1:400 clone G219-1129, Cell Marque, Rocklin, CA, USA; MSH6, dilution: 1:200 clone 44, Cell Marque, Rocklin, CA, USA and PMS2, dilution: 1:200 clone EP51, Dako, Agilent Technologies, Santa Clara, CA, USA) was performed on the four primary tumor samples. Additional stainings for Ki-67 (dilution: 1:200, clone MIB-1, Dako, Agilent Technologies Company, Santa Clara, CA, USA) and p53 (dilution: 1:700, clone Do-07 Dako, Agilent Technologies Company, Santa Clara, CA, USA) were also done. The percentage of positive cells for Ki-67 cell proliferation index and the Histo-score for p53 intensity were determined following immunostaining in the microscope. Histo-score included both the intensity of staining (graded as: 0, non-staining; 1, weak; 2, median; or 3, strong using adjacent normal mucosa as the median) and the percentage of positive cells following semi-quantitative assessment. H-score is assigned using the following formula: [1 × (% cells 1+) + 2 × (% cells 2+) + 3 × (% cells 3+)]. The range of possible scores extends from 0 to 300 [16].

Genomic DNA was extracted from FFPE tissues using QIAamp DNA FFPE Tissue Kit (Qiagen, Hilden, Germany). DNA concentration was measured in the Qubit dsDNA HS Assay Kit using a Qubit 4.0 Fluorometer (Thermo Fisher Scientific, Waltham, MA, USA).

After the fragmentation of the genomic DNA, NGS libraries were created using the TruSight Tumor 15 Kit (Illumina, San Diego, CA, USA). This panel is a targeted sequencing assay that simultaneously detects and characterizes single-nucleotide variants (SNVs), insertions and deletions (indels) in 15 genes associated with CRC tumors. The following 15 genes are included: *AKT1*, *BRAF*, *EGFR*, *ERBB2*, *FOXL2*, *GNA11*, *GNAQ*, *KIT*, *KRAS*, *KIT*, *NRAS*, *PDGFRA*, *PIK3CA*, *RET*, *TP53*. The final libraries were quantified using the Qubit 4.0 Fluorometer (Thermo Fisher Scientific, Waltham, MA, USA), diluted to a final concentration of 4 nM and pooled by equal molarity. For sequencing with the use of the MiSeq Reagent kit (v3 2 × 300 cycles), all libraries were denatured by adding 0.2 nM NaOH and diluted to 40 pM with hybridization buffer from Illumina (San Diego, CA, USA). The final loading concentration was 8 pM libraries and 1% PhiX. Sequencing was conducted according to the MiSeq instruction manual (Illumina, San Diego, CA, USA). The data were analyzed with the BaseSpase TruSight Tumor 15 Application for the presence of SNVs and indels (Illumina, San Diego, CA, USA). The sequence quality for each sample was assessed. The limit of detection was set to 2% variant allele frequency. A reliable variant detection requires coverage of >250 reads.

Reverse hybridization was carried out using *KRAS* XL StripAssay according to the manufacturer’s protocol (ViennaLab Diagnostics, Vienna, Austria). The assay covers 29 clinically relevant mutations in the *KRAS* gene and is certified for human in-vitro diagnostics (IVD). For interpretation, hybridization strips were aligned using the standardized layout supplied with the reagents and positive bands were individually identified.

For the Sanger sequencing, exon 2 of the *KRAS* gene was amplified by PCR using the forward GGTACTGGTGGAGTATTTGATAGTG and reverse CGTCAAGGCACTCTTGCCTAC primers. The purified PCR products were sequenced using the BigDye Terminator v1.1 Cycle Sequencing kit (Thermo Fisher Scientific, Waltham, MA, USA). The samples were analyzed on the ABI PRISM 310 Genetic Analyzer (Thermo Fisher Scientific, Waltham, MA, USA). VAF on sequencing electropherograms was calculated according to the following formula: mA% (proportion of mutant allele) = Hm (height of the mutant allele wave)/(Hm + Hwt (height of the wild-type allele wave))*100 [17].

## 3. Results

### 3.1. Case Presentation and Clinical Follow-Up

**Figure 1:**
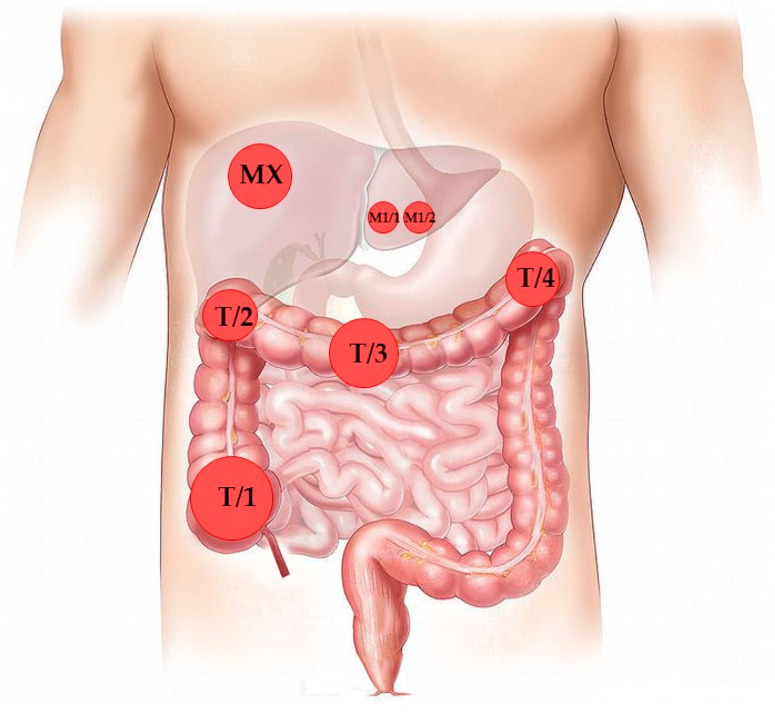
Anatomical localization of the individual primary tumors of the upper colon and of the liver metastases known at the time of the surgery. MX represents liver metastasis described by preoperative CT scan examination but removed later as M2 (hepatic VIII. segment).

A 57-year-old man with cachexia, looser stool and hematochezia was directed to the Department of Gastroenterology, University of Debrecen. The ultrasound and abdominal computer tomography (CT) scan revealed significant intestinal wall thickness and a mass of the right colon (flexura hepatica) region, moderate paraaortic lymphadenopathy and a 3.2-cm lesion corresponding to metastasis in the hepatic VI–VIII segment. The preoperative colonoscopic histology reported on a tubulovillous adenoma with focal high-grade dysplasia. Due to the imminent occlusion by the tumor, subtotal colectomy was performed. While the metastasis in the hepatic VI–VIII segment described in the CT finding could be not be approached, two smaller metastatic foci found in the hepatic III segment during surgery were removed in the same session. Detailed pathological examination of the surgical sample identified (1) a 9.5-cm moderately differentiated adenocarcinoma of the coecum, with preexisting villous polyp; (2) a 4-cm poorly differentiated adenocarcinoma of the hepatic flexure, with preexisting villous polyp; (3) an 8-cm moderately differentiated adenocarcinoma of the colon transversum, with preexisting villous polyp; (4) a 5-cm poorly differentiated adenocarcinoma of the colon transversum, without preexisting polyp; and (5) two necrotic metastatic tumors (0.8 and 1.3 cm) from the hepatic III segment with cribriform architecture (Figure 1, Table 1). The histological pattern was generally microglandular with limited masses of mucinous component. The pathologic stage was pT3, N0, M1. The preserved expression of the mismatch repair proteins MLH1, MSH2, MSH6 and PMS2 determined by IHC ruled out the major causative role of mismatch repair deficiency. Routine molecular testing of the surgical sample detected a *KRAS* mutation in the primary tumor (exon 2, codon 12 with genotype c.35G > T; p.Gly12Val). Following postoperative restaging, the presence of additional liver metastasis was stated and first-line combined chemo- and biotherapy (Folfiri and Bevacizumab) was decided on. Nearly one year later, regression determined by CT scan enabled liver exploration (M2). The patient received postoperative Xelox therapy, but the patient had severe intolerance (diarrhea and hand-foot syndrome) and the treatment was switched to Folfox. After seven months, progression was shown by CT imaging and another liver metastasis was removed, followed by postoperative deGramount therapy (M3). Nine months later, a further three independent liver metastases were removed (M4). Next, thoracic CT presented lung metastases. Based on *KRAS* wild-type status determined by Sanger sequencing from the sample M3, second-line Vectibix + Folfiri treatment was started; however, further regression could not be achieved. The last surgical intervention was the decompressive resection of a symptomatic metastatic spinal tumor (M5), which was followed by the third-line Lonsurf therapy.

### 3.2. NGS-Based Mutation Profiling

The 15-gene solid tumor panel analysis identified a series of mutations in the genes *KRAS* and *TP53*, while all other genes relevant in colon carcinoma (including *BRAF*, *EGFR* and *NRAS*) appeared to be uninvolved. As the most critical genetic change, *KRAS* status was analyzed in every detail (Table 2 *KRAS* Reference Sequence NM_004985.4.). In the preoperative colonoscopic biopsy (B), the *KRAS* variant c.35G > A; p.Gly12Asp (variant allele frequency—VAF: 10.2%) was detected, similar to the first primary tumor sample (T/1) (*KRAS* c.35G > A; p.Gly12Asp, VAF: 40.6%). In the second colon tumor sample (T/2) wild-type *KRAS* was detected. The third colon tumor (T/3) presented with the *KRAS* variant c.34G > T; p.Gly12Cys (VAF: 16.2%) and the fourth primary tumor (T/4) with the *KRAS* variant c.35G > T; p.Gly12Val (VAF: 15.1%). The hepatic metastases removed at the date of the bowel surgery presented with different genotypes: wild-type *KRAS* in the first tumor (M1/1) and the pathogenic *KRAS* variant (c.34G > T; p.Gly12Cys, VAF: 19.1%) in the second tumor (M1/2) were found. The same *KRAS* variant was confirmed (VAF: 6.4%) in the second liver metastasis (M2) resected 11 months later. In the liver metastasis from the third time point (M3, 18 months) no *KRAS* variants could be detected. Metastatic samples from the last two time points (M4, 27 months and M5, 36 months after primary surgery) presented again with the *KRAS* c.34G > T; p.Gly12Cys variant, although at very different allele frequencies (VAF: 2.0 and 32.1, respectively) (see Table 2 for details).

### 3.3. KRAS Validation Studies

**Figure 2:**
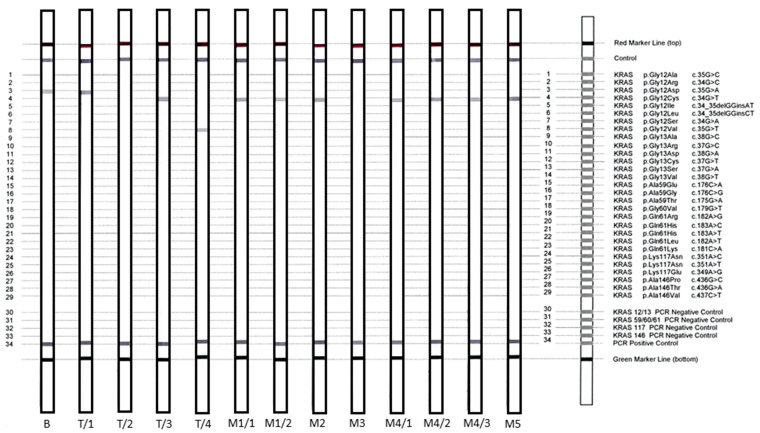
The results of a *KRAS* reverse-hybridization strip assay visually presenting *KRAS* variants as specific hybridization bands on individual membrane strips processed for all tumor samples. The adjustment of the strips to the deciphering template enables the exact determination of gene variants. Altogether, three types of codon 12 *KRAS* variants (T/1, T/3, T/4) and wild-type KRAS (T/2) were identified at the time of the primary surgery. Variant c.34G > T; p.Gly12Cys, originally present only in primary T/3, could be followed during the complete disease course, except for in sample M3 (solitary liver metastasis at month 18 from primary surgery).

**Figure 3:**
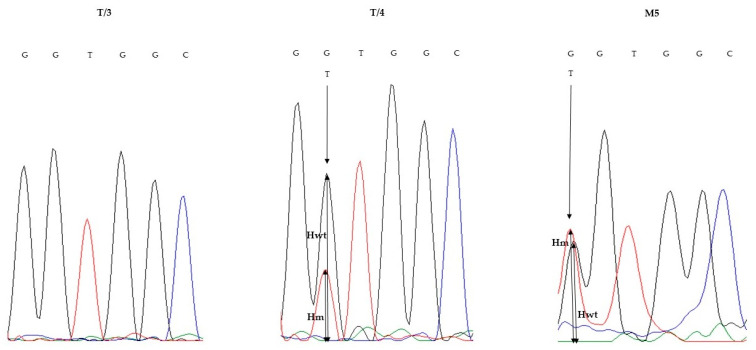
Direct sequencing (Sanger) electropherograms of codons 12 and 13 of the *KRAS* gene, demonstrating different codon 12 variants in primary tumor T/3 (wild-type, left), T/4 (c.35G > T; center) and in liver metastasis M5 (c.34G > T; right). Although T/3 proved to be *KRAS* wild-type by Sanger sequencing, low-frequency c.34G > T genotype could be safely presented using high-sensitivity NGS and StripAssay technologies. One-way arrows represent the site of the mutations and two-way arrows show the high of the waves. Hm = height of the mutant allele wave, Hwt = height of the wild-type allele wave, black wave: guanine (G), red wave: timin (T) and blue wave: cytosine (C).

Due to the complexity of the *KRAS* results obtained by NGS, we performed validation studies on *KRAS* variants at the single-gene level on two alternative platforms. The reverse-hybridization-based StripAssay constructed for IVD testing of clinically relevant *KRAS* alterations identified all variants previously detected by NGS, although quantification was not enabled (Figure 2). Classical bidirectional Sanger sequencing directed to activating *KRAS* exon 2 alterations covering codon 12/13 resulted in full agreement with the NGS data, except low-frequency alterations (below 10% VAF), which were not represented (Figure 3, Table 2).

### 3.4. TP53 Gene Sequencing and p53 sTatus

In addition to *KRAS,* the 15-gene solid tumor NGS panel indicated pathogenic *TP53* gene variants with a highly variable pattern. Altogether, 16 *TP53* variants could be detected in the 13 samples analyzed, with VAFs ranging between 2.9 and 72.8% (Table 3, *TP53* Reference Sequence NM_000546.5).

**Figure 4:**
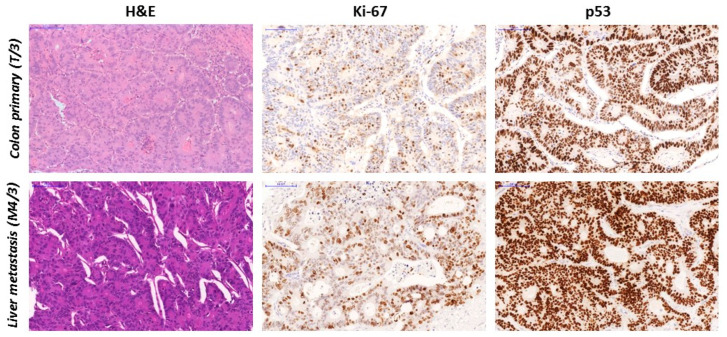
Conventional histology (H&E) and Ki-67and p53 immunohistochemistry of the primary colon adenocarcinoma sample T/3 and the late liver metastasis sample M4/3, removed 27 months after initial surgery. While the gross histological appearance (tubulo-glandular pattern with minimal foci of mucinous differentiation) of the tumor did not significantly change during the follow-up, a significant increase in cell proliferation was detected (20% vs. 40% Ki-67 labeling index, central panel). The positive p53 IHC status (generally strong positive cell nuclei, H-score = 230) of primary tumor T3 and obvious positivity (strong uniform nuclear positivity, H-score = 300) of the metastasis M4/3 (right panel) are both consistent with the respective *TP53* genotype demonstrated by NGS. Microscope magnification: 20X.

The *TP53* gene variant c.820G > T; p.Val274Phe was detected in the T/3 primary tumor and was identified in all metastatic tumors. M2 and M3 samples presented with low allele frequencies (7.1 and 3.14, respectively). In the last two metastatic samples (M4 and M5), this sequence variant was provided at highly enriched allele frequencies (72.8 and 45.6% VAF, respectively). Retrospective analysis of the p53 protein by immunohistochemistry of all histological samples resulted in different, but generally measurable degrees of immunopositivity throughout the course of the disease (Table 3). Except for the initial biopsy sample (B) and primary tumors T/1 and T/4, all samples presented with high H-scores refer to a mutant-type reaction concordant with the NGS results (Figure 4).

## 4. Discussion

Carcinogenesis in the large bowel is a heterogenous process involving several alternative cellular signaling mechanisms. The most frequent classical pathway follows the adenoma-carcinoma sequence and is highlighted by pathogenic variants of the *APC*, *KRAS, NRAS* and *TP53* genes [3,18]. In addition, CRC may evolve through the serrated–methylated pathway, which can be further subdivided into traditional and sessile serrated mechanisms according to *KRAS*/*BRAF* mutations, CpG island methylation and *MGMT* and *MLH1* methylation status [19]. CRC with germline mutations of the mismatch repair protein complex (Lynch syndrome) is considered a separate group. According to current histological practice, the exclusion of mismatch repair (MMR) deficiency is enabled by the use of an immunohistochemistry panel [20]. In addition, DNA sequencing of relevant gene segments enables the estimated categorization of the molecular subgroup, as complex genomic studies (including methylation profiling) are still unavailable for the clinical routine.

We have examined all available features to characterize the biological nature of and establish the clonal link between four simultaneously occurring primary colon tumors and eight distant metastases arising in a single patient. Based on the constant histomorphological appearance, the preserved MMR protein IHC profile, and the *KRAS*/*TP53* sequencing results, the case was interpreted as a series of conventional adenocarcinomas that developed from anatomically separated dysplastic adenomatous polyps of the right colon. The APC gene was unfortunately not covered by the gene panel applied, but the anamnesis and the clinicopathological presentation did not support familial adenomatous polyposis (FAP) etiology.

Comparison of the molecular findings clearly demonstrated the independent nature of the four primary tumors, presenting with four different KRAS genotypes, including three unrelated pathogenic *KRAS* variants (Gly12Asp, Gly12Cys, Gly12Val) and a wild-type KRAS configuration. The individual *KRAS* variants allowed the tracing of the metastatic tumors from different time points of the disease. Interestingly, a single one (T3) of the four primary adenocarcinomas could be identified as the source of all metastases occurring synchronously (M1/1 and M1/2) and during the later follow-up. The permanent exon 2, codon 12 mutant *KRAS* status (c.34G > T, p.Gly12Cys) throughout the disease also served as robust guidance for therapy selection. However, this permanent variant could not be detected in a single metastasis (M3), which does not necessarily mean the lack of that particular allele. We assume that quality variables, including tissue composition, fixation, etc., interfere with DNA-based testing, as stated by any genetic platforms applied.

Further to *KRAS*, the NGS gene panel identified diverse variants of the *TP53* gene which presented in different combinations within the individual primary tumors, also clearly justifying their clonal independence. Low-frequency (˂10%) *TP53* variants were detected in all primary samples, while dominant *TP53* changes were seen only in samples T2 and T3. However, the variant detected in T3 (c.820G > T, p.Val274Phe) was the only one that could be followed through all later samples, defining T3 primary tumor as the only source of systemic dissemination, in agreement with the *KRAS* data. Interestingly, the M3 liver metastasis featured by the wild-type KRAS result provided the constant TP53 variant, however, with a rather low allele frequency (VAF 3.14%) but a relatively high p53 H-score. In general, p53 IHC was in a good agreement with the *TP53* sequencing data, providing variable, but prominent, H-scores in T2 and T3 primary as well as in all metastatic tumors.

The comparison of diagnostic colonoscopic biopsy and the surgical material also resulted some interesting observations. The biopsy (a histologically severe form of dysplasia) revealed a *KRAS* variant (c.35G > A, p.Gly12Asp) equal to the one detected in primary tumor T1 of the surgical resection sample. According to the clinical records, colonoscopy was performed through the entire colon while the biopsy sampling was done only from the largest lesion placed in the proximal end (cecum), which is in agreement with the *KRAS* sequencing data. However, altogether, six different low-level *TP53* variants (VAF between 2.9 and 7.6) were detected in the biopsy as well as in T1, which did not show any overlap. Similar to other follow-up samples, low-frequency *TP53* variants presented by NGS appear to be sporadic and individual and do not seem to have a major effect on tumor progression.

The great advantage of NGS is the parallel detection of multiple gene variants associated with cancer progression and therapy resistance [21]. Here, we used a simple 15-gene panel covering diagnostic targets relevant for the management of CRC. This panel is affordable and efficient for sequencing in a routine setting, although the full complexity of the genome is not reflected. The reverse-hybridization-based StripAssay, on the other hand, rapidly identifies relevant hotspot mutations of solitary genes in a highly sensitive manner. The actual *KRAS* strip covers 29 specific variants for *KRAS* at a sensitivity below 1% allele frequency [22]. Finally, Sanger sequencing of solitary small regions used to be considered the “gold standard” in molecular gene diagnostics. It is still applicable for validation of DNA results from well-preserved bulky solid tumor samples, while limitations for detecting low-frequency variants (below 10% VAF) are clear.

In conclusion, the targeted sequence analysis of this unusual multiple colon cancer and related metastases provided valuable insights into the clonal relationship of the primary tumors and the genetic progression of the metastatic process treated for four years. Out of the four independent primary tumors, only one required systemic treatment due to distant metastases. Our results further emphasize the need to evaluate metastatic lesions separately for disease follow-up and therapy optimization.

## Figures and Tables

**Table 1:** Surgical interventions and histological findings and therapeutic strategy during the course of multiple metastatic colon adenocarcinoma.

Time from Primary Surgery (months)	Type of Operation	Histology	Sample ID
October 2015	Colon biopsy	Tubular adenoma with high-grade dysplasia	B
December 2015	Subtotal colectomy and liver metastasectomy	Colon adenocarcinoma (four independent sites)	T1/1-4
		Liver metastasis	M1 (M1/1, M1/2)
11	Hepatic resection	Liver metastasis	M2
18	Hepatic resection	Liver metastasis	M3
27	Hepatic resection	Liver metastasis	M4 (M4/1, M4/2, M4/3)
36	Spinal resection and decompression	Adenocarcinoma metastasis	M5

**Table 2:** *KRAS* gene variants detected by the TruSight Tumor 15 NGS panel, StripAssay and Sanger sequencing approaches from the primary tumors (T/1-4) and metastatic tumors (M1-5) throughout the course of the disease.

Samples	Tumor Cell Content (%)	NGS	NGS VAF (%)	StripAssay	Sanger Sequencing	Sanger VAF (%)
B	50	c.35G > A, p.Gly12Asp	10.2	c.35G > A, p.Gly12Asp	NMD ^b^	0
T/1	30	c.35G > A; p.Gly12Asp	40.6	c.35G > A; p.Gly12Asp	c.35G > A; p.Gly12Asp	10
T/2	50	NMD ^a^	0	NMD ^a^	NMD ^a^	0
T/3	30	c.34G > T; p.Gly12Cys	16.2	c.34G > T; p.Gly12Cys	NMD	0
T/4	30	c.35G > T; p.Gly12Val	15.1	c.35G > T; p.Gly12Val	c.35G > T; p.Gly12Val	26.86
M1/1	20	NMD ^a^	0	c.34G > T; p.Gly12Cys ^b^	NMD ^a^	0
M1/2	30	c.34G > T; p.Gly12Cys	19.1	c.34G > T; p.Gly12Cys	c.34G > T; p.Gly12Cys	45.89
M2	20	c.34G > T; p.Gly12Cys	6.4	c.34G > T; p.Gly12Cys	NMD	0
M3	30	NMD ^a^	0	NMD ^a^	NMD ^a^	0
M4/1	30	c.34G > T; p.Gly12Cys	<2	c.34G > T; p.Gly12Cys	NMD	0
M4/2		c.34G > T; p.Gly12Cys	<2	c.34G > T; p.Gly12Cys	c.34G > T; p.Gly12Cys	9.77
	30	c.35G > A; p.Gly12Asp	<2			
		c.35G > T; p.Gly12Val	<2			
M4/3	30	c.34G > T; p.Gly12Cys	5.3	c.34G > T; p.Gly12Cys	NMD	0
M5	50	c.34G > T; p.Gly12Cys	32.1	c.34G > T; p.Gly12Cys	c.34G > T; p.Gly12Cys	52

^a^ No mutation detected. ^b^ Variant detected by high-sensitivity (<1%) reverse hybridization assay.

**Table 3:** *TP53* sequencing, p53 immunohistochemistry and cell proliferation data obtained from the same tumor tissue sample. WT = wild-type, M = mutant type, P = pathogenic, N = neutral, ND = not detected, C = conflicting interpretation of pathogenicity, US = uncertain significance, LP = likely pathogenic.

Sample	NGS	NGS VAF (%)	Mitosis Index	p53 IHC (%)	p53 H-Score	p53 Interpretation	Clinical Significance (COSMIC)	Clinical Significance (ClinVar)
Biopsy	c.417G > A; p.Lys139Asn	2.9	24	2	5	WT	P	ND
	c.472C > T; p.Arg158Cys	3.8					P	C
	c.1009C > T; p.Arg337Cys	3.1					P	P
	c.464C > T; p.Thr155Ile	7.6					N	ND
T/1	c.455C > T, p.Pro152Leu	3.3	62	5	23	WT	P	ND
	c.845G > A, p.Arg282Gln	3.9					P	C
T/2	c.557C > G; p.His193Asp	35.6	71	60	155	M	ND	LP
	c.586C > T; p. Arg196*	5.6					P	P
T/3	c.820G > T; p.Val274Phe	33.3	50	90	230	M	N	LP
T/4	c.743G > A; p.Arg248Gln	3.1	62	15	25	WT	P	P
M1/1	c.726C > A; p.Cys242*	10.8	57	70	150	M	P	ND
	c.769G > A; p.Gly266Arg	7.4					ND	C
	c.328C > T; p.Arg110Cys	8.3					ND	US
	c.747G > T; p.Arg249Ser	4.0					ND	C
	c.820G > T, p.Val274Phe	23.1					N	LP
M1/2	c.827C > T; p.Ala276Val	15.7	64	100	300	M	ND	ND
	c.917G > A; p.Arg306Gln	28.1					P	C
	c.820G > T; p.Val274Phe	6.1					N	LP
M2	c.820G > T; p.Val274Phe	7.1	31	90	225	M	N	LP
M3	c.820G > T; p.Val274Phe	3.14	30	80	195	M	N	LP
M4/1	c.820G > T; p.Val274Phe	37.9	62	80	195	M	N	LP
M4/2	c.820G > T; p.Val274Phe	72.8	91	100	300	Mutant	N	LP
M4/3	c.820G > T; p.Val274Phe	25.2	46	100	300	Mutant	N	LP
M5	c.820G > T; p.Val274Phe	45.6	24	100	300	Mutant	N	LP
